# Innovating Chinese Herbal Medicine: From Traditional Health Practice to Scientific Drug Discovery

**DOI:** 10.3389/fphar.2017.00381

**Published:** 2017-06-16

**Authors:** Shuo Gu, Jianfeng Pei

**Affiliations:** ^1^Center for Quantitative Biology, Academy for Advanced Interdisciplinary Studies, Peking UniversityBeijing, China; ^2^Institute for Medical Engineering and Science, Massachusetts Institute of Technology, CambridgeMA, United States

**Keywords:** Chinese herbal medicine, traditional Chinese medicine, drug discovery, medical narrative, methodological reductionism

## Abstract

As one of the major contemporary alternative medicines, traditional Chinese medicine (TCM) continues its influence in Chinese communities and has begun to attract the academic attention in the world of western medicine. This paper aims to examine Chinese herbal medicine (CHM), the essential branch of TCM, from both narrative and scientific perspectives. CHM is a traditional health practice originated from Chinese philosophy and religion, holding the belief of holism and balance in the body. With the development of orthodox medicine and science during the last centuries, CHM also seized the opportunity to change from traditional health practice to scientific drug discovery illustrated in the famous story of the herb-derived drug artemisinin. However, hindered by its culture and founding principles, CHM faces the questions of the research paradigm posed by the convention of science. To address these questions, we discussed two essential questions concerning the relationship of CHM and science, and then upheld the paradigm of methodological reductionism in scientific research. Finally, the contemporary narrative of CHM in the 21st century was discussed in the hope to preserve this medical tradition in tandem with scientific research.

## Introduction

### The Philosophy and Ancient Narrative of Chinese Herbal Medicine

Traditional Chinese Medicine has a history of about 3000 years starting from the early Zhou Dynasty of China or even earlier as the oldest medical writings on herbs were found in *Classic of Changes* (*Yi Jing*) and *Classic of Poetry* (*Shi Jing*) (Reid, 1996). In these classics, dozens of herbs were mentioned in a variety of situations related to healing and diet. Later, TCM evolves into an independent discipline as accumulated knowledge was documented in medical books. Among which, the most famous four classics are *Inner Canon of the Yellow Emperor* (*Huang Di Nei Jing*, ∼26 BCE), *Yellow Emperor’s Canon of Eighty-One Difficult Issues* (*Nan Jing*, ∼106 CE), *Treatise on Cold Damage Disorders* (*Shang Han Lun*, ∼206 CE), and *Shennong’s Materia Medica* (*Shen Nong Ben Cao Jing*, ∼220 CE). These classic works constitute the early foundation of TCM.

While there are different branches of TCM like Chinese herbal medicine, acupuncture, and Qigong, the essential philosophy is the same. Western people might be most familiar with the metaphysics of Yin and Yang, representing the two ends of a spectrum like cold-hot, female-male, and inside-outside. When this concept is applied to the human body, Yin and Yang are linked to different parts or organs of the body, or simply one’s feeling of cold and hot. Consistent with the philosophy of Taoism, one is expected to keep balance of his Yin and Yang; otherwise the breaking of balance gives rise to different syndromes or diseases. In such cases, herbs with “hot” properties can be used to treat a “cold” syndrome, and vice versa (Chan, 1995).

Another fundamental theory in TCM is the Five Elements theory, which describes the human body and herbs with five elemental qualities (Wood, Fire, Earth, Metal, and Water). For example, Water represents kidney, bladder, and ears, while Fire represents heart, small intestine, and tongue. Furthermore, since the five elements are associated in both the generating circle (Wood -> Fire -> Earth -> Mental -> Water -> Wood) and the overcoming circle (Wood -> Earth -> Water -> Fire -> Mental -> Wood), all the organs in the body are also connected and can be affected according to the attributes of certain herbs (Chan, 1995).

Before the 20th century, most Chinese could only seek TCM as a medical service, in which herbal medicine was intensively used. The history of Chinese herbal medicine (CHM) is largely associated with the creative yet appealing narratives of healing. The narrative tradition, either in the form of classics or passed down orally, preserves the medical knowledge of ancient Chinese people and continues to strengthen the belief in herbal healing in the Chinese communities to this day. It appears that the majority of Chinese still believe in the power of CHM, although orthodox medicine is popular and affordable in China now. Similar to a religion, CHM in China is almost like a faith in the herbal materials that can cure disease and save life.

Then, how did the ancestors of Chinese link various herbs to certain diseases? One possible reason could be the doctrine of signatures that Chinese people tend to relate the efficacy of a herb to its name, shape, or other figurative notions (Bennett, 2007). For example, ginseng in Chinese means “essence of men.” People also call it “earth elf” because the shape of a ginseng root resembles a little man. In the medical usage, only the root is chosen as the medicinal material because the ancient Chinese believed this figurative part to help restore the inner energy of the human body (Hu, 1977). Moreover, the older the ginseng root, the better quality it is assumed as the longevity of a man-shaped herb can increase the life expectancy of the consumer. Although scientific findings do not necessarily support the argument, this traditional belief is quite popular in China. Put another way, the ancient narratives of CHM dominate one’s understanding toward herbs and consequently strengthen the belief in herbal healing.

### In Comparison with the Treatments in Medieval Medicine of Western Europe

The Medieval medicine of western Europe originated from ancient Greek medicine. Greek people believed that the universe is composed of four elements (fire, air, water, and earth), while the human body is also made of these: fire for liver, air for heart, water for brain, and earth for spleen. Meanwhile, there are four primal qualities (warm, cold, dry, and moist) describing the attributes of each element, e.g., warm and dry for fire.

In one medieval medical book *Causes and Cures* written by Hildegard of Bingen, we can find interesting treatments whose logic is quite similar to that of CHM. “If persons cannot hold urine due to the coldness of their stomachs, they should frequently drink wine that has been heated over fire, mix vinegar into all their foods and drink vinegar as often as possible. This way the stomach and bladder will be warm” (Berger, 1999). Another example is about female infertility. “A woman whose uterus is too cold within and too weak to conceive offspring can, if God wills, be aided to be fertile in the following way: take the uterus of either a lamb or a cow that is sexually mature but still pure in that it is not and has not been with young. Cook it with lard, other fatty meat and fat, and give that to the woman to eat when she has or will very soon have intercourse with her husband” (Berger, 1999).

As we compare the treatments from traditional West and East, both are associated with the imagination of the human body and medicinal materials. Before orthodox medicine revealed the real cause of disease, human beings had a long history of creative imagination about the inner and outer self. While ancient Chinese perceived the body in an adequate balance characterized by Yin and Yang, medieval European doctors treated a patient’s coldness with the wine heated by fire. Since the hot/cold dichotomy is quite widespread in folk medicine, the principles of treatments are unsurprisingly the same (Foster, 1978). For example, similar to the way that the man-shaped ginseng is used to restore the inner energy of body, the uterus of either a lamb or a cow was also used for female infertility in medieval Europe.

## Discussion

### Innovating Chinese Herbal Medicine

The 19th century witnessed the rise of modern medicine, nourished by science and technology during the same time. Since then, orthodox medicine became a scientific discipline supported by rigorous biomedical studies. However, it was until 20th century that TCM was reexamined through the lens of science. During the past decades, numerous studies were carried out by various methods in order to demystify the power of curative herbs (Normile, 2003; Tan and Vanitha, 2004; Jiang, 2005; Wang et al., 2006; Wang and Xiong, 2012).

Along with the persistent efforts of several generations of scientists, CHM indeed impressed the world with an effective therapy for people suffering from malaria. This drug is artemisinin, derived from *Artemisia annua* (Qinghao in Chinese), also known as sweet wormwood. During the 1960s, a group of scientists in China were assembled under a military project to find a treatment for malaria. Youyou Tu was one of them. After examining more than 2000 herbs, she narrowed down the targets to about 640. In 1971, Tu and her co-workers started to focus on herb Qinghao which showed promising inhibition to the parasite. However, this exciting observation couldn’t repeat in the following experiments. Luckily enough, Tu found a medical classic by Ge Hong (284–346 CE). In *A Handbook of Prescriptions for Emergencies*, Ge wrote: “A handful of Qinghao immersed with two liters of water, wring out the juice and drink it all.” Different from a typical decoction of herbs, this unique preparation inspired Tu that heating would probably destroy the structure of the bioactive molecules. Soon, they modified the procedure to reduce the extraction temperature by using ethyl ether. The new sample of Qinghao ether extract showed 95–100% inhibition of rodent malaria. After this success, they began to isolate and purify the bioactive molecules in the extract. In 1972, an antimalarial compound was isolated with a formula of C_15_H_22_O_5_, which was further crystalized and determined with the stereostructure in 1974. After the chemical structure was revealed, Tu and her co-workers studied its structure-efficacy correlation, which eventually led to the invention of dihydroartemisinin with improved efficacy (Tu, 2011, 2016). Artemisinin and its derivative dihydroartemisinin saved millions of lives threatened by malaria throughout the world. This discovery was considered one of the breakthroughs in human health during the last century. Due to her tremendous contributions, Tu was awarded the 2011 Lasker Award for clinical research and the 2015 Nobel Prize in Physiology or Medicine (Neill, 2011; Su and Miller, 2015).

The story of *A. annua* and Youyou Tu tells us the best outcome of herbal medicine in the context of science, that through the rigorous biomedical research, the healing mechanism of a herb was revealed from the ancient medical narratives. In other words, scientists are innovating CHM using scientific concepts and techniques, which provide us with a new understanding on this traditional health practice. Such techniques can be analytical chemistry (Jiang et al., 2010; Tistaert et al., 2011), systems biology (Wang et al., 2005, 2009), network pharmacology (Li et al., 2011; Li and Zhang, 2013), and computational modeling (Lukman et al., 2007; Barlow et al., 2012), with which, CHM is no longer what people perceived before World War II. It’s approached with modern concepts, techniques, and methods, in the way that it can be appreciated by people all over the world.

### Two Essential Questions from the Relationship of CHM and Science

When studying CHM, researchers usually argue if CHM is a science or can we apply scientific methods in the investigation. As a mixture of Chinese philosophy, culture, ritual and medical practices, CHM demands a comprehensive understanding which is not restricted to science. However, it doesn’t prevent us from studying herbs by scientific methods and evaluating the performance of herbal medicine scientifically. In order to gain a contemporary understanding of CHM, we either seek the assistance of science or adopt a novel narrative approach (which will be discussed in the last section). Science bears its value in an unbiased standard that may fulfill CHM in the global setting just like artemisinin, which is no longer considered as herbal medicine but a universal antimalaria therapy acknowledged in and out of Chinese communities.

Another essential question: is it necessary for the scientific research of CHM to be guided by the records in medical classics? In the Chinese communities, we hear voices like the traditional narratives of herbs are still valid in the context of science; therefore, we need to make efforts to embody the ancient wisdom with scientific findings, as what Youyou Tu achieved in discovering artemisinin. While such an argument holds in limited scenarios, it’s again not necessary to restrict science to these traditional writings. For example, although artemisinin was originally discovered as an antimalarial drug, recent studies show it also demonstrates anti-inflammatory, immunoregulatory, and anticancer functions which were not documented in any medical classics (Nakase et al., 2008; Shi et al., 2015). Neither do men put new wine into old bottles. Basically speaking, drug discovery in CHM is totally different from the knowledge in medical classics. In those classics, there is no modern concept of drug – molecules that can cure diseases. Furthermore, the concept of disease in the ancient Chinese context also bears a different notion far from what we understand in orthodox medicine. In the scientific study of the herbs, researchers can simply treat them as plants without any prior knowledge of healing potential because such narratives were not evaluated in any rigorous experiments and can be guiding and misleading at the same time. Therefore, to achieve an unbiased research of CHM, it’s encouraged to put aside the medical classics although such information might offer some hints.

### The Paradigm of Holism and Methodological Reductionism

Traditionally, a prescription of CHM is a unique formula tailored for the patient. In the formula, herbs are combined in a hierarchy of Principal, Associate, Assistant, and Coordinator. The herbs are also characterized according to their nature (hot, warm, cool, and cold) and flavor (acrid, sweet, bitter, sour, and salty), which need careful combination in the prescription (Chan, 1995). Doctors of TCM frequently advocate the philosophy of holism in medical practices as they hold firmly that everything is interconnected. Simply put, one’s disease or syndrome is associated with various organs governed by the Five Elements theory. Therefore, the treatment as a prescription should also address different parts of the body. For example, a famous formula for the common cold is composed of four herbs: *Coptis chinensis*, *Scutellaria baicalensis*, *Phellodendron amurense*, and *Gardenia florida* (documented by Wang Dao), while the first three are responsible for clearing the heat at heart, lung, and kidney, respectively (Zeng et al., 2011).

Following the traditional practices of herbal medicine, some scientists adopt the paradigm of holism in drug discovery of CHM, as they believe this paradigm can best appreciate the ancient wisdom of herb combination (Leung et al., 2014). It’s natural that if we stick to the traditional narratives of CHM, the formula should be evaluated as a whole and any separation inevitably results in decrease or even void in its performance. Ideologically speaking, holism distinguishes CHM as a unique health practice. However, while emphasizing the narrative on wholeness of herb and body, one is likely to neglect the building components of herbal medicine and their detailed mechanisms with drug targets.

Based on our standpoints on the two essential questions addressed above, we suggest methodological reductionism to be adopted in drug discovery of CHM. In order to acknowledge the holistic nature of herb formula, it’s a prerequisite to study the building blocks for understanding the complex system. To be concrete, here we would like to propose a three-step roadmap. First, it’s fundamental to know the molecular elements in herbal medicine. For each herb, scientists are encouraged to identify as many molecules as possible and organize the information in databases. Secondly, we are obliged to clarify the toxicity and bioactivity of the identified molecules as it lays the foundation for future pharmaceutical study of the natural products. These two steps are also understood as reverse pharmacognosy for accelerating natural drug discovery (Do and Bernard, 2004). Finally, in order to reveal the mechanisms of a herb or formula, one can test the combinatorial function of composing compounds in the biological networks with respect to human diseases (Gu et al., 2013a,b). With that, the holistic practices in CHM can be revealed at the systemic level and engineered in a scientific manner. After a comprehensive understanding being achieved, we can further apply engineering approaches like systems biology to design tailored formulas targeting specific biological networks or diseases.

### The Contemporary Narrative of Chinese Herbal Medicine in the 21st Century

However, as a double-edged sword, science also threatens CHM while fulfilling it because by the rigid scientific standards, the medical narratives of herbs are too vulnerable. Scientists and physicians, after rigorous examination, may discover an abundance of controversies between the facts and the classical writings. Especially during the past decades, CHM has been severely challenged on its efficacy and safety (Qiu, 2007). As a result, how can we appreciate herbal medicine in tandem with orthodox medicine? If we narrate CHM in the postmodern world full of scientific quests and proofs, what else can we offer other than the medical classics and scientific research? It is actually a very difficult question to answer; yet it is vital for the next generation of Chinese to keep their medical traditions.

As we have learned from our history, narrative is powerful because it keeps the identity of an ethnic group, transcending the collective memories into belief. Therefore, the questions on efficacy and safety raised by scientific research no longer hold back the faith in herbal medicine because CHM should not be judged only by facts, but also be appreciated by either individual or collective memories of the patients. A good example is the placebo effect (Kaptchuk, 2002). In order to construct the contemporary narratives of CHM, we suggest to alter the narrative subject from physicians to patients. Different from the orthodox medicine, CHM has a long history of practices in the homology of medicine and food. Herbs are never pure medicine in the Chinese context – they can be tonic or simple food. This special tradition lays the foundation for consumers to be the narrative subject, although their voices were neglected previously. Throughout the history, medical knowledges is usually documented or inherited by doctors, while the narratives of patients are undervalued (Charon, 2001). In the postmodern time, we hope to include patients as the narrator. Platforms like a forum could be a choice where patients can write to share their personal experiences of taking certain herbs in the treatment of certain diseases. With such a platform, people share their own clinical information of CHM, which can further be evaluated by researchers. Patients, both as the subject of disease and medical receiver, are appropriate and able to undertake the role of narrator, offering his own assessment of this alternative medicine, if not orthodox medicine, which requires professional knowledge to articulate (Greenhalgh and Hurwitz, 1999). With that, the narratives of CHM can be more comprehensive and robust in the 21st century.

## Conclusion

In most current studies of Chinese herbal medicine, researchers examine the efficacy and safety of herbs from a scientific perspective. However, given the importance of its cultural and religious essence, the treatments of CHM are largely associated with the traditional narratives. After World War II, a group of Chinese scientists began to study CHM using scientific methods and achieved tremendous success in the discovery of artemisinin. As a result, CHM was innovated in the form of scientific drug discovery. However, this journey is not easy as we need to overcome the old ideology inherited from the history of TCM. The holistic principles of CHM also raise many debates. By addressing the two essential questions in the relationship of CHM and science, we upheld the paradigm of methodological reductionism and further proposed a three-step roadmap of drug discovery in herbal medicine. Finally, besides the scientific perspective, we suggested the contemporary narratives of CHM be shifted from physician based to patient based for the purpose of preserving this medical tradition as well as the ethnic identity.

## Author Contributions

SG and JP conceived the study and wrote the manuscript together.

## Conflict of Interest Statement

The authors declare that the research was conducted in the absence of any commercial or financial relationships that could be construed as a potential conflict of interest.
